# Notes and News

**Published:** 1900-06-09

**Authors:** 


### Notes and News.

Mr. Nathaniel Catchpole has handed to the East Suffolk and Ipswich Hospital the sum of £1,000 for the endowment in perpetuity of a bed in the institution. This endowment is the seventh received by the institution since the addition of the Jubilee Memorial Wing was decided upon.

The arrangements for foreign members of the International Medical Congress in Paris promise to be practical and convenient. Visitors of each separate nationality will receive tickets of the same colour, so that a countryman will be quickly recognised, and interpreters and guide books will be available for all.

The feeble-minded children who are under the care of the Metropolitan Asylums Board are to be taught marketing. They are, in the terms of the resolution, to be employed in making purchases of every day articles, a most practical bit of education. But why the feeble-minded only? Perhaps in the future we shall see the children at our pauper schools going out to buy the workhouse dinner.

The people in New York are feeling very seriously what one may call the secondary difficulties connected with overcrowding. Everyone recognises the evils arising from crowded rooms; but when instead of houses being three or, at the most, four storeys high they run up to nearly twice that height, the question of space in the streets becomes a very important one. As the *Medical News* remarks, it has come not to be a question of cubic space in bedrooms and in living-rooms, but of ground breathing space and elbow-room; for in many of these neighbourhoods the street is the only playground, and when six layers of budding humanity come to be discharged into them every morning the situation becomes serious. As it seems that in New York these tall, insanitary types of buildings, which rely upon delusive air-shafts as a source of light and air, are being built at the rate of about two thousand a year, it is evident that some action must be taken in the matter, and that the Tenement-house Commission has been appointed none too soon. Those who wish to be convinced of the necessity of all town dwellings being erected under the stringent provisions of a carefully-drawn Building Act should pay a visit to New York.

The College of Surgeons hold its centenary at the end of the present month. A series of gatherings is arranged at the Mansion House, Lincoln's Inn Fields, and University College. Many distinguished foreigners have been invited to attend.

An innovation in provincial hospital procedure has been made by the authorities of the Royal Portsmouth Hospital, who have given a hospital dinner in the Mayor's Banqueting Hall, Portsmouth, for the benefit of the institution. The dinner was largely attended, and raised £800.

Shetland is to have a hospital through the generosity of the late Miss Inga Bain and her sister, Mrs. Mathew Anderson. Miss Bain made a bequest for the purpose of establishing a hospital, which has been supplemented by a gift of £2,000 towards both building and endowment, which warrants the pursuance of the scheme. The hospital is to be called the Gilbert Bain Hospital.

Some time hence we pointed out the misplaced zeal which was being exhibited throughout the United Kingdom in establishing at no inconsiderable outlay homes for convalescent soldiers returning from South Africa. We pointed out that considerable difficulties stood in the way of lay authorities, even if soldiers were willing to delay the return to their homes when convalescent. Our contentions have proved correct. At Needwood, near Bournemouth, for example, a convalescent home was established through the efforts of sympathisers in the locality, but more especially through the generosity of one lady who lent her house for the purpose. In money £696 was subscribed. Eighteen men were received, six of whom were almost immediately discharged for bad conduct. Of the money received £126 was actually spent. It was decided to close the home as further patients were not expected. The surplus money contributed will be distributed later.

The annual dinner of the Medical Graduates' College and Polyclinic was held last week at the Trocadero Restaurant. Lord Strathcona presided; and among those present were: Mr. Jonathan Hutchinson, Mr. Brudenall Carter, Sir Joseph Fayrer, Mr. Sydney Holland, Canon Ainger, Sir Henry Burdett, Dr. Theodore Williams, Dr. Stephen Mackenzie, Sir J. Batty Tuke, M.P., Dr. Seymour Taylor, and others. In proposing the toast of the evening, the Chairman, after describing the objects of the institution, said that it not only aimed at bringing together for further instruction those who had graduated in medicine or surgery, so that they might keep abreast of the advance of the science of their profession, but it brought within its door practitioners from all parts of the United Kingdom, from abroad, and from the different outlying parts of the Empire. Speaking of the Colonial members of the profession, he expressed the hope that we should some day have one of the profession extending to all parts of the Empire. Mr. Hutchinson, in proposing "The Hospitals and Kindred Institutions," disclaimed all thought of any sort of competition between the Polyclinic and the general hospitals. They were anxious, he said, to be co-workers with kindred institutions, and to send those who were associated with the Medical Graduates' College into the wards of all the great hospitals.

